# NANOS2 is a sequence-specific mRNA-binding protein that promotes transcript degradation in spermatogonial stem cells

**DOI:** 10.1016/j.isci.2021.102762

**Published:** 2021-06-24

**Authors:** Azzurra Codino, Tomasz Turowski, Louie N. van de Lagemaat, Ivayla Ivanova, Andrea Tavosanis, Christian Much, Tania Auchynnikava, Lina Vasiliauskaitė, Marcos Morgan, Juri Rappsilber, Robin C. Allshire, Kamil R. Kranc, David Tollervey, Dónal O'Carroll

**Affiliations:** 1 Centre for Regenerative Medicine, Institute for Stem Cell Research, School of Biological Sciences, University of Edinburgh, 5 Little France Drive, Edinburgh EH16 4UU, UK; 2 Wellcome Centre for Cell Biology, School of Biological Sciences, University of Edinburgh, Edinburgh EH9 3BF, UK; 3 Institute of Biotechnology, Technische Universität Berlin, Berlin, Germany; 4 Laboratory of Haematopoietic Stem Cell & Leukaemia Biology, Centre for Haemato-Oncology, Barts Cancer Institute, Queen Mary University of London, London EC1M 6BQ, UK

**Keywords:** Biological sciences, Molecular biology, Developmental biology, Omics, Transcriptomics

## Abstract

Spermatogonial stem cells (SSCs) sustain spermatogenesis and fertility throughout adult male life. The conserved RNA-binding protein NANOS2 is essential for the maintenance of SSCs, but its targets and mechanisms of function are not fully understood. Here, we generated a fully functional epitope-tagged *Nanos2* mouse allele and applied the highly stringent cross-linking and analysis of cDNAs to define NANOS2 RNA occupancy in SSC lines. NANOS2 recognizes the AUKAAWU consensus motif, mostly found in the 3′ untranslated region of defined messenger RNAs (mRNAs). We find that NANOS2 is a regulator of key signaling and metabolic pathways whose dosage or activity are known to be critical for SSC maintenance. NANOS2 interacts with components of CCR4-NOT deadenylase complex in SSC lines, and consequently, NANOS2 binding reduces the half-lives of target transcripts. In summary, NANOS2 contributes to SSC maintenance through the regulation of target mRNA stability and key self-renewal pathways.

## Highlights

• NANOS2 is a 3′UTR mRNA binding protein and recognizes the AUKAAWU consensus
• CCR4-NOT deadenylase complex is the primary interactor of NANOS2 in spermatogonia
• NANOS2 binding results in mRNA destabilization
• NANOS2 regulates key spermatogonial signaling and metabolic pathways

## Graphical abstract

**Figure:**
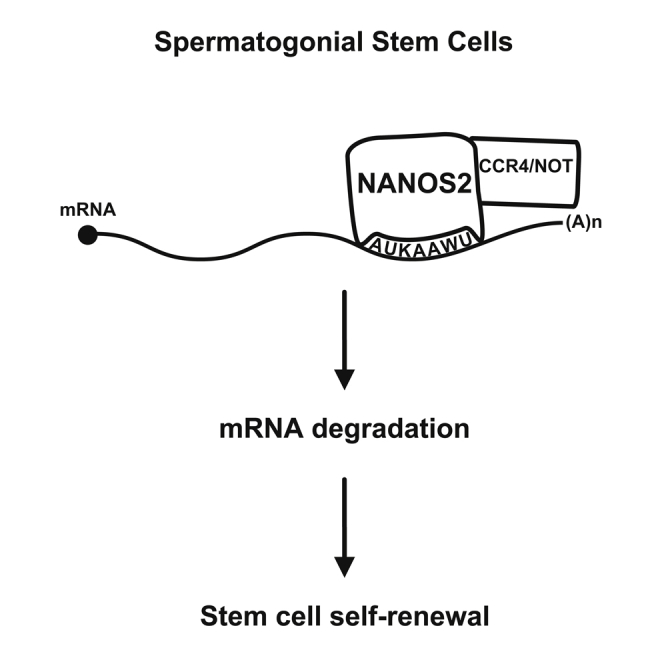

## Introduction

The maintenance of spermatogonial stem cells (SSCs) is essential to sustain life-long spermatogenesis and adult male fertility. In mouse, this population of stem cells resides within undifferentiated type A spermatogonia (Huckins, 1971; Oakberg, 1971). Undifferentiated spermatogonia comprise A_single_ (A_s_ or isolated cells) and A_paired_ (A_pr_, a pair of connected cells) or A_aligned_ (A_al_, chains of 4, 8, or 16 connected cells) cells that arise due to incomplete cytokinesis and remain attached by intercellular bridges. The glial-cell-line-derived neurotrophic factor (GDNF) receptor Gfrα1 marks a subset of A_s_, A_pr_, and A_al4_ that contains SSCs (Hara et al., 2014; Nakagawa et al., 2010). Gfrα1-positive A_s_ are heterogeneous and include a subset of cells expressing ID4 and PAX7, which have SSC activity (Aloisio et al., 2014; Chan et al., 2014; Helsel et al., 2017; Sun et al., 2015). Upon Gfrα1 downregulation, SSCs give rise to an intermediate population of spermatogenic precursors, marked by Ngn3, which are responsive to retinoic acid (RA) signaling and thus can differentiate into c-Kit-positive cells (Ikami et al., 2015; Nakagawa et al., 2010). Mechanistically, RA instructs spermatogonia differentiation through the activation of the PI3K/AKT/mTORC1 signaling pathway, which in turn enhances the translation of differentiation-related genes such as c-Kit (Busada et al., 2014, 2015a, 2015a; 2015a). SSC self-renewal is extrinsically dependent upon growth factors, such as GDNF and fibroblast growth factor (FGF) (Kitadate et al., 2019; Meng et al., 2000). Furthermore, GDNF, FGF2, epidermal growth factor (EGF), and leukemia inhibitory factor (LIF) are key to promote SSC maintenance in long-term cultures (Kanatsu-Shinohara et al., 2003; Kubota et al., 2004; Takashima and Shinohara, 2018). Downstream signaling cascades activated by these growth factors, such as phosphatidylinositol 3-kinase (PI3K)-Akt and ERK/MAPK pathways are also essential for SSC self-renewal (Hasegawa et al., 2013; Ishii et al., 2012; Lee et al., 2007). The signaling cascades activated by the aforementioned growth factors input into the mTOR pathway (Laplante and Sabatini, 2012; Liu and Sabatini, 2020; Yu and Cui, 2016). Tight control of mTOR complex 1 (mTORC1) activity is critical in regulating the balance between self-renewal and differentiation in SSCs (Busada et al., 2015b; Hobbs et al., 2010; Serra et al., 2017), as shown by the progressive spermatogenic failure associated with the alteration of the mTORC1 component RAPTOR or the regulatory TSC1/2 complex (Hobbs et al., 2015; Serra et al., 2019; Wang et al., 2016).

The RNA-binding protein (RBP) NANOS2 is mostly expressed in A_s_ and A_pr_ spermatogonia (Suzuki et al., 2009) and is required for the self-renewal of SSCs (Sada et al., 2009). Conditional ablation of *Nanos2* in adult testis results in the rapid depletion of undifferentiated type A spermatogonia and the progressive loss of spermatogenesis, whereas the overexpression of *Nanos2* results in the accumulation of undifferentiated type A spermatogonia (Sada et al., 2009). *Nanos2* is also required for the survival of male mouse gonocytes during embryonic development (Tsuda et al., 2003), where one function of NANOS2 is to supress aberrant entry into meiosis (Suzuki and Saga, 2008). In summary, NANOS2 is a key intrinsic regulator of the male germline.

*Nanos2* encodes a protein of 136 amino acids that contains two CCHC type zinc fingers and belongs to the family of NANOS RBPs with evolutionary conserved functions in the germline (De Keuckelaere et al., 2018; Tsuda et al., 2003). In mouse male embryonic gonocytes, NANOS2 interacts with the CCR4-NOT deadenylation complex and may promote transcript degradation in P-bodies (Suzuki et al., 2010). Moreover, CNOT1-NANOS2 interaction is essential for NANOS2 function during embryonic development (Suzuki et al., 2012). In postnatal SSCs, NANOS2 has been linked to the translational repression of several transcripts encoding proteins associated with SSC differentiation (Zhou et al., 2015b). NANOS2 via protein-protein interaction also sequesters mTOR in cytoplasmic messenger ribonucleoproteins to limit its activity (Zhou et al., 2015b). However, stringent biochemical approaches have not been employed yet to identify the full complement of *bona fide* NANOS2 RNA targets in SSCs, raising the possibility that additional and overlooked molecular mechanisms can underlie NANOS2 function in mouse SSCs.

## Results

### Generation and validation of the epitope-tagged *Nanos2*^*TAG*^ allele

**Figure 1:**
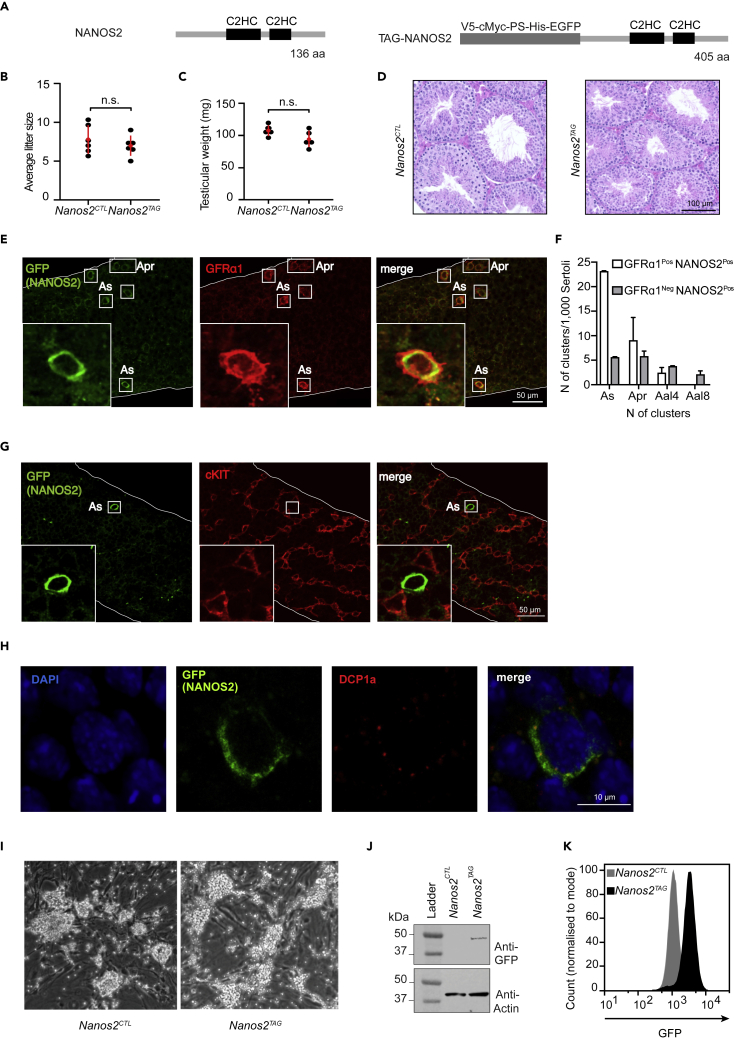
The *Nanos2*^*TAG*^ mouse allele is functional (A) Schematic representation of the NANOS2 protein and the TAG-NANOS2 fusion protein, with corresponding lengths of amino acid sequences (aa). (B) Number of litters per animal from *Nanos2*^*CTL*^*(Nanos2*^*+/+*^*)* and *Nanos2*^*TAG*^ (*Nanos2*^*TAG/TAG*^) mice. Data are mean and s.d., *n* = 6 for both genotypes. n.s. indicates not significant, p value > 0.05 using two-tailed Student's *t*-test. (C) Testicular weight of *Nanos2*^*CTL*^ and *Nanos2*^*TAG*^ from six-months-old mice. Data are mean and s.d., *n* = 5 for both genotypes. Statistical evaluation as in panel b. (D) Representative testis cross-sections stained with hematoxylin and eosin (H&E) from six-months-old *Nanos2*^*CTL*^ and *Nanos2*^*TAG*^ mice. Scale bar, 100 μm. (E) Representative immunofluorescent images of *Nanos2*^*TAG*^ seminiferous tubules stained with anti-GFP (green) and anti-GFRα1 (red) antibodies. Representative examples of GFRα1^Pos^, NANOS2^Pos^ cells (A_s_ and A_pr_) are highlighted (white boxes). Scale bar, 50 μm. (F) Enumeration of GFRα1^Pos^ NANOS2^Pos^ and GFRα1^Neg^ NANOS2^Pos^ testicular populations. The number of cells (N) present in each cluster is normalized to 1,000 Sertoli cells (y axis). Error bars represent SEM (standard error of mean). (G) Representative immunofluorescent images of *Nanos2*^*TAG*^ seminiferous tubules stained with anti-GFP (green) and anti-c-KIT (red) antibodies. Representative example of NANOS2^Pos^, c-KIT^Neg^ cell (A_s_) is highlighted (white box). Scale bar, 50 μm. (H) Representative immunofluorescent image of an A_s_ cell from *Nanos2*^*TAG*^ seminiferous tubules stained with with DAPI (blue), anti-GFP antibody (green) and anti-DCP1a (red). Scale bar, 5 μm. (I) Representative bright field images of *Nanos2*^*CTL*^ and *Nanos2*^*TAG*^ SSC lines cultured on mouse embryonic fibroblasts (MEF) feeders. (J) Western blot using anti-GFP and anti-Actin antibodies on *Nanos2*^*CTL*^ and *Nanos2*^*TAG*^ SSC lines lysates. (K) Flow cytometry analysis of *Nanos2*^*CTL*^ and *Nanos2*^*TAG*^ SSC lines. The GFP intensity of the *Nanos2*^*TAG*^ population is represented in a single-parameter histogram, with the overlay of the *Nanos2*^*CTL*^ control.

To explore the function of NANOS2 in SSCs, we generated an endogenously tagged allele of *Nanos2* (*Nanos2*^*TAG*^) in the mouse. We inserted a complex tag that consisted of the V5 tag, the Myc tag, the precision protease site, the His tag, and the enhanced green fluorescent protein (EGFP) that generates an N-terminal NANOS2 fusion protein (Figures 1A and S1). This versatile tag enables many applications including immunofluorescence and immunoprecipitation approaches. Homozygous *Nanos2*^*TAG*^ mice were fertile and presented normal testicular weight as well as seminiferous tubule histology (Figures 1B–1D). Despite the size of the tag, the lack of phenotype in *Nanos2*^*TAG*^ mice demonstrates the functionality of the epitope-tagged allele. This genetic complementation is in stark contrast to the impact of *Nanos2* deficiency. *Nanos2* is essential for both the development of the male gonocytes and self-renewal of SSCs (Sada et al., 2009; Tsuda et al., 2003). In the adult testis *Nanos2* expression is restricted to undifferentiated spermatogonia, many of which are GFRα1-positive (Sada et al., 2009; Suzuki et al., 2009). We used anti-GFP antibodies to examine the expression of TAG-NANOS2 in whole-mount preparations of seminiferous tubules, which confirmed the restricted cellular expression profile. TAG-NANOS2 expression was restricted to undifferentiated spermatogonia, most of which were Gfrα1 positive (Figures 1E and 1F). Indeed, all Gfrα1-positive spermatogonia expressed TAG-NANOS2 (Figure 1F), as was previously reported for endogenous NANOS2 (Suzuki et al., 2009). PLZF staining that marks a wide population of progenitor spermatogonia from A_s_ to very long chains revealed that TAG-NANOS2 expression is restricted predominantly to A_s_ and A_pr_ rather than longer spermatogonial chains (Figure S2). Importantly, TAG-NANOS2 was not identified in c-Kit-positive differentiating spermatogonia (Figure 1G). Similar to what has been reported for endogenous NANOS2 (Sada et al., 2009; Suzuki et al., 2009; Zhou et al., 2015b), TAG-NANOS2 is localized to the cytoplasm and also found in P-bodies (Figure 1H). In summary, we conclude that the *Nanos2*^*TAG*^ allele is functional and faithfully recapitulates the reported expression of *Nanos2*.

The transcriptome occupancy or the complement of transcripts bound by NANOS2 as well as a possible consensus binding site for NANOS2 remains uncharacterized by stringent cross-linking immunoprecipitation techniques coupled to high-throughput sequencing (CLIP-seq) approaches. RNA immunoprecipitation (RIP) of NANOS2 coupled with microarray hybridization from P7 testis (Zhou et al., 2015b) or from E14.5 fetal testis (Saba et al., 2014) had previously been used to define NANOS2 target transcripts. However, RIP is the least stringent method for target identification because the RNA is not cross-linked to the RBP and low-stringency washes can only be applied. In the case of NANOS2, this method is more problematic given that a portion of NANOS2 resides in P-bodies (Suzuki et al., 2010; Zhou et al., 2015b), and as such, NANOS2 RIP may readout many RNAs found therein. Furthermore, because cross-linking is not applied prior to the lysis of cells, RIP-based approaches are prone to artifacts due to the mixing of cellular compartments and the reassembly of new complexes with nonphysiological targets (Lee and Ule, 2018; Mili and Steitz, 2004). One limitation of CLIP-seq is the requirement of large amounts of input material due to the low efficient UV cross-linking of RNA to RBPs (Ramanathan et al., 2019). This constraint precludes the application of CLIP-seq from *ex vivo* isolated NANOS2-expressing spermatogonia. Fortunately, SSC lines derived from neonatal testis (Kanatsu-Shinohara et al., 2003) can be expanded in culture for prolonged periods of time, retaining their spermatogonial identity (Kanatsu-Shinohara et al., 2003). These cultures display functional heterogeneity as only a small fraction of these cells have the ability to produce colonies in recipient mouse testis in transplantation experiments (Kanatsu-Shinohara and Shinohara, 2013). They can also display heterogeneity in terms of different spermatogonia marker expression (Kanatsu-Shinohara et al., 2003). Nonetheless SSC cultures represent a valuable surrogate system for the study of SSCs and spermatogonia *in vitro* (Kanatsu-Shinohara and Shinohara, 2013). Thus, we derived control *Nanos2* wild type (*Nanos2*^*CTL*^) and experimental *Nanos2*^*TAG/TAG*^ or *Nanos2*^*TAG/+*^ (*Nanos2*^*TAG*^*)* SSC lines from postnatal testis (Figure 1I). All cell lines, independent of genotype, displayed the expected morphology (Figure 1I). TAG-NANOS2 could be detected by Western blotting from the SSC lines (Figure 1J), and more importantly, the *Nanos2*^*TAG*^ cell lines uniformly expressed TAG-NANOS2 as determined by FACS analysis using the fused EGFP (Figure 1K).

### CRAC reveals NANOS2 transcriptome occupancy in SSC lines

**Figure 2:**
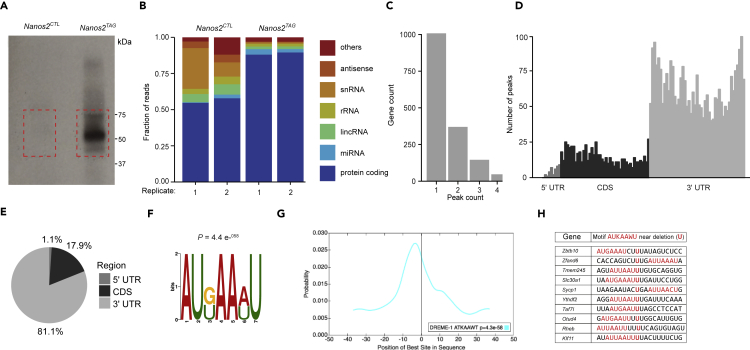
CRAC reveals NANOS2 occupancy, consensus binding sequence and preference for mRNA 3′ UTR residency in SSC lines (A) Autoradiograph showing the TAG-NANOS2 protein purified from homozygous *Nanos2*^*TAG*^ SSC lines by SDS-PAGE and cross-linked to radioactively labeled RNA. Dashed boxes indicate the area excised for the elution of RNA-protein complexes. (B) Transcriptomic distribution of mapped CRAC reads among different classes of RNAs as indicated is shown. (C) The number of NANOS2 peaks in individual mRNAs identified within sequencing reads is shown. (D)Metagene profile of NANOS2 CRAC peaks along the 5′ UTR, CDS, and 3′ UTR of its targets. The average length of these regions is shown. (E) Percentages of NANOS2 peaks within 5′ UTR, CDS, and 3′ UTR are shown. (F) Logo representation of the NANOS2-binding site present in the top 500 NANOS2 targets using reads with deletions. The logo is adapted from MEME-ChIP. (G) Position of the AUKAAWU motif within sequencing reads (adapted from Centrimo). Reads were centered on deletions. (H) Sequence alignment of a subset of reads with deletions, from the top 500 NANOS2 targets. The AUKAAWU motif is highlighted in red. Sequences are centered on deletions, which correspond to the cross-linking sites, and the most probable NANOS2 direct binding site (central ‘U’, in bold red).

To gain insight into the mechanism by which NANOS2 maintains SSC self-renewal, we aimed to define NANOS2 transcriptome occupancy in SSCs by using cross-linking and analysis of cDNAs (CRAC) (Granneman et al., 2009). CRAC is one of the most stringent methods to identify high confident RNA-protein interactions due to the fact that only RBPs and RNAs at zero distance are cross-linked and that the cross-linked-RBP-RNA complexes are purified in multiple steps, with the last two being under denaturing conditions (Granneman et al., 2009; Lee and Ule, 2018; Ramanathan et al., 2019) (Figure S3). The *Nanos2*^*TAG*^ SSC lines permit the use of CRAC thanks to the presence of the V5 epitope tag, the PreScission protease recognition site and the His6 tag within TAG-NANOS2 (Granneman et al., 2009). After TAG-NANOS2 was UV-C cross-linked to RNAs in SSCs, TAG-NANOS2-RNA complexes were sequentially purified and analyzed by autoradiography. Coprecipitated RNA was recovered from *Nanos2*^*TAG*^ but not *Nanos2*^*WT*^ SSC lines (Figure 2A). RNA was isolated and converted into cDNA to generate libraries for high-throughput sequencing. After mapping sequencing reads to the mouse transcriptome, we analyzed the similarities among replicates and samples by calculating the Spearman's correlation coefficient. *Nanos2*^*TAG*^ replicates highly correlated with each other, while no correlation was observed between *Nanos2*^*TAG*^ and *Nanos2*^*CTL*^ (Figure S4A), indicating the specificity of the CRAC experiments. CRAC analysis revealed that NANOS2 predominantly bound (>88%) to mRNA (Figure 2B; Table S1), generally with one binding site per transcript (Figure 2C). Importantly, the vast majority of NANOS2-biding sites (81%) were located in the 3′ UTRs of transcripts (Figures 2D and 2E). For the transcripts with multiple NANOS2 binding sites, the length of the 3′UTR was not a determinant of the number of binding sites (Figure S4B).

The sequence information from CRAC experiments can be used to define the binding site of RBPs (Granneman et al., 2009). UV cross-linking generally results in covalent binding of a nucleotide (most commonly a pyrimidine) to one amino acid residue of the RBP (Sugimoto et al., 2012). The sites of amino acid-nucleotide cross-linking are problematic for reverse transcriptases. The enzyme can terminate at those positions or readthrough by overcoming the cross-linked amino acid, potentially incorporating an error or microdeletion into the nascent cDNA (Granneman et al., 2009; Ule et al., 2003, 2005). Among mutations contained in read through cDNA, single-nucleotide deletions reflect most accurately the cross-linking site, since these are very rarely generated through PCR or sequencing errors (Granneman et al., 2009; Zhang and Darnell, 2011). Thus, we analyzed CRAC reads with deletions to precisely identify NANOS2-binding sites. We first normalized CRAC-binding site intensities for transcript expression levels, as determined by RNA-seq. This approach is commonly used as CRAC read counts are partially dependent on the expression level of the respective transcripts present in cells (König et al., 2012). Comparing the top 500 normalized CRAC TAG-NANOS2-bound transcripts in SSC lines (Table S2), we found a significant overlap with the RIP-identified NANOS2 target transcripts from P7 testis and E14.5 fetal testis (Zhou et al., 2015b; Saba et al., 2014) (Figures S4C and S4D, Tables S3 and S4). MEME-ChIP analysis (Machanick and Bailey, 2011) was performed on the top 500 most highly enriched transcripts (Table S2) that contained deletions. This identified a putative consensus, seven-nucleotide-binding site for NANOS2 (AUKAAWU; with K = G or U, W = A or U) (Figure 2F), that was significantly enriched in the proximity of deletions (p = 4.4 x 10^−58^) (Figures 2G, 2H, and S4E). In summary, in SSCs, NANOS2 predominantly binds mRNAs, generally at a single site in the 3′UTR, with a preference for the consensus sequence AUKAAWU.

### NANOS2 regulates many transcripts important for SSC metabolism and self-renewal

**Figure 3:**
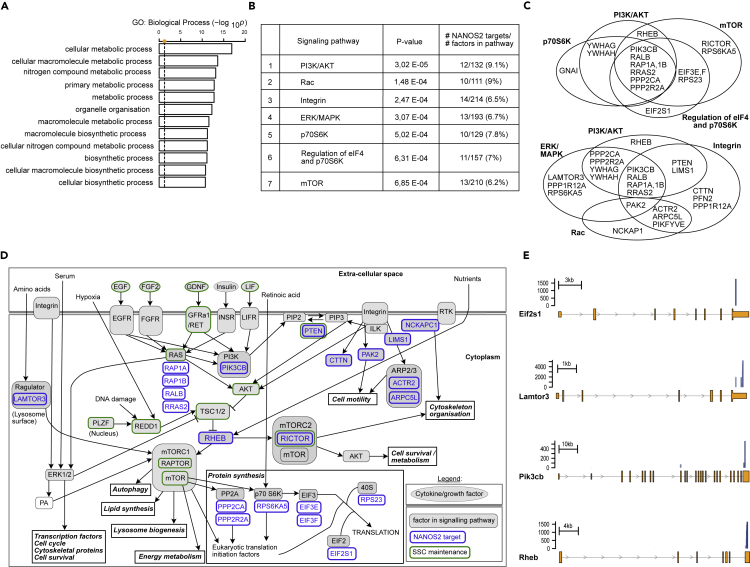
NANOS2 binds to mRNAs encoding proteins involved in metabolic and signaling pathways critical for SSC maintenance (A) Gene ontology (GO) analysis performed on the top 500 NANOS2 CRAC targets. The significance of enrichment of Biological Processes was calculated by Fisher's exact test and is shown as -log_10_ p value. The dashed line shows the threshold for the significance (p value < 0.05). (B) Ingenuity pathways enrichment analysis performed on the top 500 NANOS2 CRAC targets. The enrichment of signaling pathways is shown in the second column (p value < 0.05). The number and percentage of NANOS2 targets in each pathway are also shown (third column). (C) Overlapping NANOS2 targets within the seven signaling pathways are shown as indicated. (D) The scheme shows some of the molecular relationships within these pathways with NANOS2 targets in blue, and factors critical for SSC maintenance in green. (E) Representative examples of NANOS2 peaks in four mRNA targets (*Eif2s1, Pik3cb, Lamtor3* and *Rheb*) encoding proteins involved in the signaling pathways described in (B and C).

Having defined transcripts bound by NANOS2, we next sought to understand how this contributes to the maintenance of SSCs. Gene ontology analysis of the top 500 NANOS2-bound transcripts revealed very significant enrichment for metabolic and biosynthetic processes (Figure 3A). We next applied an Ingenuity Pathway Analysis (Krämer et al., 2014), which revealed enrichment for seven signaling pathways: PI3K/AKT, RAC, Integrin, ERK/MAPK, p70S6K, regulation of eiF4 and p70S6K as well as mTOR (Figure 3B). These pathway classifications were consistent with the identification of many specific NANOS2 target mRNAs implicated in the respective pathways (Figure 3C). Importantly, this analysis included pathways whose activity and regulation are known to be essential for the maintenance of SSCs (Hasegawa et al., 2013; Hobbs et al., 2010; Ishii et al., 2012; Lee et al., 2007; Oatley et al., 2007) or for spermatogonia proliferation (Feng et al., 2000): PI3K/AKT, ERK/MAPK, p70S6K, and mTOR (Figure 3D). Besides its role in SSC maintenance (Goertz et al., 2011; Oatley et al., 2007), PI3K/AKT is also involved in mediating spermatogonia differentiation in cells responsive to RA (Busada et al., 2015b). Many of these signaling pathways are directly stimulated by growth factors which are also essential for SSCs. SSCs are absolutely dependent on GDNF – Gfrα1 signaling (Kanatsu-Shinohara et al., 2003; Oatley et al., 2007). Indeed heterozygosity of GDNF results in the progressive loss of SSCs whereas its overexpression results in expansion of SSCs (Meng et al., 2000). FGF signaling is also important for the self-renewal of SSCs as is EGF (Ishii et al., 2012; Kanatsu-Shinohara et al., 2003; Kitadate et al., 2019; Takashima et al., 2015). Among NANOS2 targets, were mRNAs encoding key signaling transduction molecules, as exemplified by the RAS-related proteins RAP1A/B, RALB, and RRAS2 and the PIK3CB and PTEN proteins. These factors act downstream of the GDNF, FGF, EGF receptor tyrosine kinases (Figures 3C–3E) and, importantly, RAS and PTEN are essential for SSC self-renewal (Lee et al., 2009; Zhou et al., 2015a). These growth factors signaling pathways input into the mTOR pathway (Meng et al., 2018), which regulates cell growth and metabolism (Laplante and Sabatini, 2012). Moreover, mTOR activity supports SSC differentiation at the expenses of self-renewal (Busada et al., 2015b; Hobbs et al., 2010; Wang et al., 2016). Notably, the mTORC2 component RICTOR is essential for spermatogonia differentiation (Bai et al., 2018), whereas the mTORC1 component RAPTOR and the mTORC1 negative regulator TSC1/2 are essential for the maintenance of the SSC pool (Hobbs et al., 2015; Serra et al., 2019; Wang et al., 2016). Interestingly, we identified the transcripts for the mTORC2 component RICTOR and mTORC1 regulators RHEB and LAMTOR3 as NANOS2 targets (Figures 3C–3E). Furthermore, many transcripts that encode proteins downstream of mTOR in the regulation of protein synthesis are targets of NANOS2, as exemplified by PPP2CA, PPP2R2A, RPS6KA5, EIF3E/F, RPS23, and EIF2S1 (Figures 3D and 3E). In summary, a subset of NANOS2 target transcripts encompasses key components of signaling and metabolic pathways whose regulated activity is known to be essential for SSC self-renewal (Hobbs et al., 2015; Lee et al., 2009; Serra et al., 2019; Wang et al., 2016; Zhou et al., 2015a).

### NANOS2 binds the CCR4-NOT complex and reduces mRNA half-lives in SSCs

**Figure 4:**
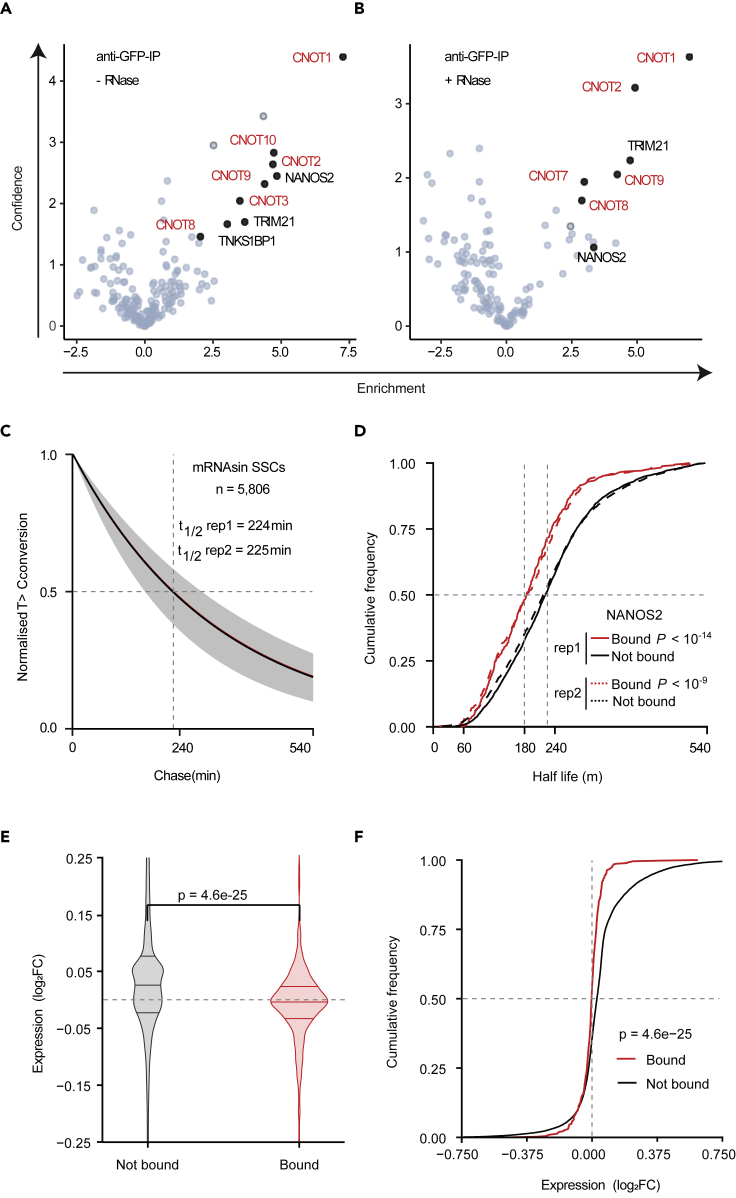
NANOS2 interacts with CNOT proteins and its binding to mRNA is associated with transcript destabilization in SSC lines (A and B) Volcano plots showing results from the immunoprecipitation (using anti-GFP beads) followed by mass spectrometry analysis from *Nanos2*^*CTL*^ and *Nanos2*^*TAG/TAG*^ SSC lines. IPs were treated without (−) (A) or with (+) (B) RNase as indicated. Each TAG-NANOS2 interactor is shown as a dot and significantly enriched proteins are labeled (except for NANOS2 in B). The x axis shows the enrichment of the interactors in the IPs (log2(LFQ(Nanos2^TAG^)/LFQ(Nanos2^WT^); the y axis shows the confidence (-log_10_(p value of two-sided Student's *t*-test)). CNOT proteins are highlighted in red. Each plot shows results from three replicates (N = 3). (C) Median decay curves showing the global mRNA stability in SSC lines for two replicates. mRNA half-life (t_1/2_) was determined for 5,806 transcripts by counting the T to C conversion rate in sequencing reads, over time (minutes). The shaded areas indicate the first and third quantile decay curves range for each replicate. Transcript half-life median for each replicate are indicated with horizontal dotted lines and are also shown at the panel top. (D) Cumulative distribution of mRNA half-life in replicate 1 (rep1) (solid lines) and 2 (rep2) (dashed lines) for NANOS2 targets. ‘Bound’ targets, in red, are top 500 NANOS2 CRAC targets; ‘Not bound’, in black, are mRNAs not bound by NANOS2. The indicated significance of difference between the NANOS2-bound and unbound transcripts was computed by Mann-Whitney U test. (E) Violin plots showing expression log_2_ fold change in *Nanos2* overexpressing compared to wild-type SSCs. Putative top-500 NANOS2-bound transcripts (annotated “Bound”) are compared to transcripts lacking CRAC peaks (annotated “Not bound”). The upper and lower quartiles and the median are indicated for each group. p value for group difference computed by Mann-Whitney U test. (F) Cumulative distribution plots showing log_2_ fold expression change in *Nanos2* overexpressing cells. Transcript groups are as in (E) above: top-500 NANOS2-bound (“Bound”) transcripts compared to unbound transcripts (“Not bound,” lacking NANOS2-CRAC peaks). p value for group difference computed by Mann-Whitney U test.

We next sought to understand the mechanism by which NANOS2 regulates its target transcripts in SSCs. CRAC revealed that the majority of NANOS2 binding occurs within the 3′ UTR of mRNAs. 3′ UTRs are frequently bound by protein complexes that can modulate mRNA translation, deadenylation, and decay (Mayya and Duchaine, 2019). To understand which of these processes were involved in the regulation of NANOS2 targets, we aimed to identify NANOS2 interacting proteins in SSCs. To this end, we performed immunoprecipitation coupled to mass spectrometry (IP-MS) from SSC lines (Figure 4A). Using stringent criteria of greater than four times enrichment and significance of p value < 0.05, we found 8 factors (Figure 4A, Table S5). Among these were 6 subunits of the CCR4-NOT deadenylase complex, including the catalytic subunits CNOT7 and CNOT8. NANOS2 interaction with the CCR4-NOT complex was independent of RNA, as treatment of the IP with RNase A and T1 still resulted in the enrichment of many CCR4-NOT subunits (Figure 4B, Table S5). The CCR4-NOT complex is the major cytoplasmic deadenylase and mRNA deadenylation constitutes the first, rate-limiting step of RNA degradation (Bresson and Tollervey, 2018). These results suggest that NANOS2 recruits the CCR4-NOT complex to its mRNA targets and stimulate their degradation. To test this, we performed SLAM-seq (Herzog et al., 2017) in SSC lines, which enables the measurement of mRNA half-life transcriptome-wide. SLAM-seq analysis revealed that the average transcript half-life is 224–225 min (Figure 4C). Strikingly, NANOS2-bound transcripts had a much shorter half-life (180 min) relative to transcripts that were not bound by NANOS2 (Figure 4D). NANOS2 is essential for SSC maintenance, whereas its overexpression is compatible with spermatogonial survival albeit with the loss of their ability to differentiate (Sada et al., 2009). Thus, unlike *Nanos2*-deficiency, its overexpression is not required for SSC line survival (Zhou et al., 2015b). NANOS2 overexpression in SSC lines (Zhou et al., 2015b) resulted in an overall reduction in the abundance of NANOS2 target transcripts relative to other mRNAs (Figures 4E and 4F). We conclude that in SSCs, NANOS2 recruits the CCR4-NOT deadenylase complex to mRNAs, which in turn promotes a reduction in transcript half-life.

## Discussion

We have generated a versatile epitope-tagged *Nanos2* allele that has enabled the molecular exploration of its function in SSCs. Notably, the use of transcriptomics and proteomics methods allowed us to explore NANOS2 mechanism of function in SSCs. The application of the stringent CRAC defined the NANOS2-bound target mRNAs transcriptome-wide. It also revealed that mouse NANOS2 directly binds RNA and has a well-defined consensus sequence, AUKAAWU. The length of the consensus is consistent with RNA binding by both zinc fingers of NANOS2, since each is predicted to recognize three nucleotides (Choo and Klug, 1994). *Drosophila* NANOS does not bind RNA on its own, but only in cooperation with PUMILIO (Sonoda and Wharton, 1999; Weidmann et al., 2016). First, PUMILIO binds mRNAs through an eight-nucleotide consensus motif, and NANOS subsequently joins the complex (Murata and Wharton, 1995; Sonoda and Wharton, 1999; Wharton and Struhl, 1991). After that, PUMILIO consensus changes by enriching for A/U elements upstream of its binding site (Weidmann et al., 2016). In contrast, our data reveal that mouse NANOS2 can directly bind RNA and also with sequence-specificity. Thus, despite NANOS proteins being highly conserved across evolution, the mode of NANOS2 binding has diverged from invertebrates to mammals. This conclusion is supported by the fact that the mammalian PUM2 is not required for mouse fertility (Xu et al., 2007) and that PUM1, although it is expressed throughout spermatogenesis, does not have a defined spermatogonial function (Chen et al., 2012). Furthermore, our protein-interaction studies in SSCs did not identify PUM1/2 or any other RBP as NANOS2 partners and the NANOS2-PUM1/2 interaction was not observed from E15.5 (Suzuki et al., 2016). This supports the model that NANOS2 is sufficient for the binding and selection of its RNAs targets in mouse SSCs.

Our data showed that NANOS2 mainly binds mRNAs within their 3′ UTRs, suggesting that NANOS2 could regulate the fate of its mRNA targets through post-transcriptional mechanisms (Mayya and Duchaine, 2019). Indeed, we found that many components of CCR4-NOT deadenylase complex copurified with NANOS2 in extracts from SSC lines. Association of NANOS2 with the CCR4-NOT complex was previously reported in embryonic gonads (Suzuki et al., 2010), and NANOS2 was shown to interact with CNOT9 by co-IP in SSCs (Zhou et al., 2015b). Moreover, the interaction between NANOS2 and CNOT1 is essential for the development of male germ cells (Suzuki et al., 2012). *In vitro* deadenylase assays showed that immunoprecipitated NANOS2 from testis can deadenylate RNA substrates (Suzuki et al., 2010), suggesting that NANOS2 may promote transcript degradation. Importantly, by using a transcriptome-wide approach to measure mRNA half-life in SSC lines, we have now demonstrated that NANOS2 binding is associated with an average 20% reduction of transcript half-life, which across its many targets is likely to significantly impact the proteome. We therefore propose that in addition to the translational and sequestering mechanisms previously presented (Zhou et al., 2015b), an important mechanism of NANOS2 function in SSCs is to promote transcript turnover.

By employing CRAC, we have now defined the full complement of NANOS2-bound transcripts in SSC lines. The SSC cultures contain a mixture of SSCs and progenitor cells, and as such, the NANOS2 target transcripts identified will be from both spermatogonial stages. We did observe a significant overlap between our study and the RIP-based studies form E14.5 and P7 testis (Saba et al., 2014; Zhou et al., 2015b) (Figures S4C and S4D, Tables S2 and S3), which provides an independent *in vivo* validation of our approach. The differences in NANOS2 targets between the various datasets likely arise from the methodology used as well as the cell type examined, which differed between all the respective studies. The NANOS2-binding sites defined herein also provide a more thorough understanding of the genome through the possible interpretation of single-nucleotide polymorphisms or mutations associated with male infertility. It was previously shown by RIP that NANOS2 binds specific mRNAs encoding proteins important for SSC differentiation, including *Sohlh2*, *Dazl* and *Taf7l* (Zhou et al., 2015b). CRAC also revealed many other NANOS2 targets, which are strongly over-represented for mRNAs encoding proteins involved in cellular metabolism and biosynthetic processes. The precise regulation of cellular metabolism is key for spermatogenesis (Rato et al., 2012) and also for SSC self-renewal (Kanatsu-Shinohara et al., 2016; Morimoto et al., 2015). Many adult stem cells, including SSCs, must tightly control their proliferation rate in order to prevent premature exhaustion of the stem cell pool over time. By sensing and integrating multiple growth signals, mTOR balances many biosynthetic processes and thus sustains the anabolic growth and proliferation (Ben-Sahra et al., 2013; Kim and Guan, 2019; Laplante and Sabatini, 2012). Indeed, mTOR signaling stimulates spermatogonia proliferation and differentiation (Busada et al., 2015b; Feng et al., 2000; Hobbs et al., 2015), and consistently, long-term maintenance of SSCs requires that mTOR activity be retained at minimal levels (Hobbs et al., 2010; Wang et al., 2016). Importantly, we found that NANOS2 targets are enriched for mRNAs encoding proteins involved in mTOR signaling and other pathways which are essential for the regulation of SSC self-renewal. We conclude that selective binding by NANOS2 regulates the half-life of key mRNA targets, which are directly and indirectly involved in the control of the metabolic status and growth of the cell. In summary, we propose that NANOS2 represses many targets in order to regulate SSC quiescence and protect self-renewal potential.

### Limitations of the study

We have identified NANOS2-bound transcripts by using CRAC, a stringent biochemical method, in mouse SSC lines. However, we could not directly validate these NANOS2-RNA interactions *in vivo* by the same methodology due to the limiting amount of material that can be obtained from *ex vivo*-isolated spermatogonial populations. However, many of these CRAC-identified transcripts were also found as NANOS2 targets in NANOS2-RIP experiments performed in mouse embryonic and postnatal testes (Saba et al., 2014) (Zhou et al., 2015b), suggesting that SSC lines represent a good surrogate for their *in vivo* counterparts. Among NANOS2-bound transcripts identified by CRAC, we found many mRNAs involved in metabolic pathways which were previously shown to be important for SSC self-renewal. However, in this study, we have not investigated the contribution of NANOS2-mediated regulation of individual target transcripts to SSC maintenance. In the future, it will be interesting to see if mutations within NANOS2 consensus sites in target genes are associated with male infertility in mouse models or in humans.

## STAR★Methods

### Key resources table

**Table:** 

REAGENT or RESOURCE	SOURCE	IDENTIFIER
**Antibodies**		

Mouse monoclonal anti GFP (Clones 7.1 and 13.1)	Roche	Cat#11 814 460 001; RRID: AB_390913
Mouse monoclonal anti V5	Invitrogen	Cat#R960-CUS; RRID: AB_2556564
Chicken Polyclonal anti GFP	Aves	Cat#GFP-1010; RRID: AB_2307313
Goat anti GFRα1	Neuromics	Cat#GT15004; RRID: AB_2307379
Goat Polyclonal α-GFP	ThermoFisher	Cat#A-11122; RRID: AB_221569
Goat polyclonal anti C-kit	R&D Systems	Cat#AF1356-SP
Rabbit polyclonal anti PLZF	Santa Cruz Biotechnology	Cat#sc-22839; RRID: AB_2304760
Rabbit polyclonal anti GFP	ThermoFisher	Cat#A11122; RRID: AB_221569
Mouse monoclonal anti Dcp1a	Sigma-Aldrich	Cat#WH0055802M6; RRID: AB_1843673

**Chemicals, peptides, and recombinant proteins**		

TAG-NANOS2 (N-terminal tagged NANOS2 protein)	This paper	N/A

**Critical commercial assays**		

SLAMseq Explorer Kit – Cell viability Titration Module	Lexogen	Cat#SKU: 059.24
SLAMseq Kinetics Kit – Catabolic Kinetics Module	Lexogen	Cat#SKU: 062.24
QuantSeq 3′ mRNA-Seq Library Prep Kit FWD	Lexogen	Cat#SKU: 015.24
SENSE Total RNA-Seq Library Prep Kit	Lexogen	Cat#SKU: 042.24
Ni-NTA Superflow	QIAGEN	Cat#30410
RNace-It Ribonuclease cocktail	Agilent	Cat#400720
RNasin Ribonuclease Inhibitor	Promega	Cat#N2115
Pierce spin columns snap cap	Thermo Scientific	Cat#69725
MetaPhor agarose	Lonza	Cat#50180
LA Taq	Takara	Cat#RR002M
MinElute Gel Extraction kit	QIAGEN	Cat#28604

**Deposited data**		

CRAC-seq	This paper	GEO accession: GSE149835
RNA-seq	This paper	GEO accession: GSE149835
SLAM-seq	This paper	GEO accession: GSE149835
NANOS2 protein interactome in SSCs (IP-Mass spectrometry)	This paper	Table S5

**Experimental models: Cell lines**		

Mouse spermatogonial stem cells: *Nanos2*^*CTL*^	This paper	N/A
Mouse spermatogonial stem cells: *Nanos2*^*TAG/+*^	This paper	N/A
Mouse spermatogonial stem cells: *Nanos2*^*TAG/"tnTAG*^	This paper	N/A
Mouse embryonic fibroblasts (feeders for SSCs)	This paper	N/A

**Experimental models: Organisms/strains**		

Mouse: *Nanos2*^*TAG*^ DBA/2J x C57Bl/6 hybrid	This paper (DBA/2J strain from Charles River Laboratories)	DBA/2J strain code: 625
Mouse: *Nanos2*^*TAG*^ C57Bl/6	This paper	N/A

**Recombinant DNA**		

V5-c-Myc-PS-His6X-EGFP-FRT-neo-FRT (PS is PreScission)	This paper	N/A

**Software and algorithms**		

FastQC software	http://www.bioinformatics.babraham.ac.uk/projects/fastqc/	N/A
FASTX-collapser v0.0.14)	http://hannonlab.cshl.edu/fastx_toolkit/	N/A
pyCRAC	(Webb et al., 2014)	https://bitbucket.org/sgrann/pycrac
flexbar v3.4.0	(Dodt et al., 2012)	N/A
STAR v. 2.5.3a aligner	(Dobin et al., 2013)	N/A
Salmon v. 0.13.1 quasi-quantification	(Patro et al., 2017)	N/A
HTSeq v.0.11.2	(Anders et al., 2015)	N/A
bamCoverage v3.1.3 (deepTools package)	(Ramírez et al., 2016)	N/A
SAMtools v1.9	(Li et al., 2009)	http://www.htslib.org/
Novoalign v2.07.00	Novocraft	http://www.novocraft.com
Integrative Genomics Viewer	Broad Institute	http://software.broadinstitute.org/software/igv/; RRID:SCR_011793
python 2.7 Jupiter notebooks, python libraries (pandas v0.19.2, NumPy v1.16.0, scipy v1.2.0)	(Turowski et al., 2016).	update of gwide toolkit v0.5.27 https://github.com/tturowski/gwide
Bioconductor Limma Package	(Ritchie et al., 2015)	https://bioconductor.org/packages/release/bioc/html/limma.html
Bioconductor topGO package	Bioconductor	https://bioconductor.org/packages/release/bioc/html/topGO.html
stats R package	R project	https://www.r-project.org/
MEME-ChIP	Machanick and Bailey, 2011	https://meme-suite.org/meme/tools/meme-chip
SlamDunk pipeline	(Neumann et al., 2019)	https://github.com/t-neumann/slamdunk
MaxQuant LFQ algorithm	(Cox et al., 2014)	N/A
Perseus version 1.6.0.2	(Tyanova et al., 2016)	N/A
ingenuity-pathway-analysis IPA (QIAGEN Inc.)	(Krämer et al., 2014)	https://www.qiagenbio-informatics.com/products/ingenuity-pathway-analysis
Affinity designer	https://affinity.serif.com/en-gb/	N/A
FlowJo software	https://www.flowjo.com/solutions/flowjo/downloads	N/A
Fiji ImageJ.	https://imagej.net/Downloads	N/A

### Resource availability

#### Lead contact

Further information and requests for resources and reagents should be directed to and will be fulfilled by the lead contact, Dónal O’Carroll (donal.ocarroll@ed.ac.uk).

#### Materials availability

Reagents generated in this study are available upon request from the Lead Contact.

#### Data and code availability

The CRAC, RNA-seq, and SLAM-seq data sets generated in this study are available at GEO, accession GSE149835. The data supporting this study are available in the supplementary information.

### Experimental models and subject details

#### Mouse generation and maintenance

For the generation of the *Nanos2*^*TAG*^ allele, the sequence encoding V5-c-myc-PreScission-6xHis-EGFP (named ‘TAG’) was inserted after the endogenous ATG start codon of *Nanos2.* The targeting construct including the TAG sequence was genetically modified to include homology arms and an FRT-flanked neomycin cassette 3' of the 3' UTR. Individual A9 ESC clones were screened for homologous recombinants by Southern blotting of NheI-digested DNA with a 3' probe. Southern blotting was performed as described (Morgan et al., 2017). The *Nanos2*^*WT*^ locus generated a DNA fragment of 6.6 kb, whereas the targeted *Nanos2*^*TAG-neo*^ locus produced a 3.8 kb fragment due to the presence of an additional NheI site, introduced after the integration of the 3′ FRT-flanked neomycin cassette in the *Nanos2*^*TAG-neo*^ locus. To generate mice A9-targeted ESCs were injected into C57BL/6 eight-cell-stage embryos, as described (De Fazio et al., 2011). *Nanos2*^*TAG-neo*^ mice were crossed to FLP-expressing transgenic mice (FLPeR (Farley et al., 2000)) to induce FLP-mediated excision of the *FRT*-flanked neomycin cassette, thus generating *Nanos2*^*TAG*^ mice. Female and male *Nanos2*^*TAG*^ mice were maintained on the C57BL/6 genetic background. For SSCs derivation, female *Nanos2*^*TAG*^ mice were crossed with DBA/2J male mice (purchased from the Charles River Laboratories) to generate mice in a mixed background. Female and male *Nanos2*^*WT*^ and *Nanos2*^*TAG*^ alleles were genotyped by PCR after extracting genomic DNA from ear biopsies using the following primers: “FW1_N2”: 5'-AACCTGGGGAATAACCTGCT-3', “FW2_N2”: 5'-TGCTGCTGAATAAAGCGTTG-3', “RV_N2”: 5'-TCCCAGTCAGACGACTTGTG-3'. Fertility of male mice was assessed by setting up matings with 2 months old C57Bl/6 females and litter size was analyzed for the following 3-4 months, at pups’ weaning age.

Mice were generated at the EMBL Mouse Biology Unit, Monterotondo, and later bred and maintained at the Scottish Centre for Regenerative Medicine, University of Edinburgh. All procedures were done in accordance with the Italian legislation (Art. 9, 27. Jan 1992, nu116) or under UK Home Office authorization.

#### Derivation and maintenance of SSC lines

SSCs were derived from testis of postnatal day 7 mice (males) and maintained as described in (Kanatsu-Shinohara et al., 2003), with some modifications to the media. Cells were cultured in the following cell media (SSC media): Stem Pro-34 SFM medium with Supplements (2,6 %), Invitrogen (10639-011); 6 mg/ml D-(+)-Glucose, Sigma (G7021); 25 μg/ml Insulin, Sigma (I5500); 5 ml/ml BSA, MP-Biomedicals (810661); 100 μg/ml apo-Transferrin human (diluted in Stem Pro-34 SFM medium), Sigma (T1147); 30 nM Sodium Selenite (diluted in Stem Pro-34 SFM medium), Sigma (S5261); 1X MEM Vitamin solution, Invitrogen (11120037); 10 μg/ml D-Biotin (diluted in Stem Pro-34 SFM medium), Sigma (B4501); 60 ng/ml Progesterone (diluted in 100% Ethanol), Sigma (P8783); 30 ng/ml β-estradiol (diluted in 100% Ethanol), Sigma (E2758); 1X Sodium Pyruvate, Sigma (P2256-5G); 60 μM Putrescine (diluted in Stem Pro-34 SFM medium), Sigma (P7505); 2 mM L-Glutamine, Invitrogen (25030-024); 5.7 10^-7^ M β-Mercaptoethanol, Bio-Rad (1610710); 1 μl/ml Lactate, Sigma (L4263); 1% FBS (HyClone Fetal Bovine Serum Characterized, 12379802, Fisher Scientific); 20 ng/ml epidermal growth factor (EGF), (EMBL); 10 ng/ml, fibroblast growth factor 2 (FGF2), (EMBL); 10 ng/ml glial cell line-derived neurotrophic factor (GDNF), (EMBL); 20 ng/ml Leukemia Inhibitory Factor (LIF), (EMBL); 100 U/ml Penicillin-Streptomycin, Invitrogen (15140-122). Cytokines were produced at the ‘Protein Expression & Purification’ Core facility, EMBL Heidelberg, Germany. SSCs were frozen in 50% FBS (12379802), 40% SSC media, 10% DMSO (D2650, Sigma). Both MEFs and SSCs were cultured at 37°C and 7.5% CO2 and were tested for mycoplasma contamination routinely.

### Method details

#### Histology

Testes were fixed in Bouin’s solution (16045-1, Polysciences) and embedded in paraffin. Testes were sectioned with a microtome to obtain 4-μm-thick slices, every ∼300-400 μm, to have ∼15 sections for testis. Testes slices were obtained from the beginning, a quarter and half of the testis longitudinal length. Slices were placed on a glass slide and these were stained with hematoxylin and eosin (H&E), according to standard protocols. Images of tubules’ sections were acquired with Zeiss Axio Scan Slide Scanner and the presence of spermatogenic cells was evaluated by analyzing images with the Zen Airyscan and Fiji ImageJ softwares.

#### Whole-mount immunofluorescence on seminiferous tubules

Whole-mount immunofluorescence of seminiferous tubules was performed as described (Carrieri et al., 2017). For the expression analysis of TAG-NANOS2 and GFRα1, co-staining of tubules was done with two primary antibodies: α-GFP, 1:500 (GFP-1010, Aves), α-GFRα1, 1:50 (GT15004, Neuromics). For the expression analysis of TAG-NANOS2 and c-KIT, co-staining was done with: α-GFP, 1:200 (A-11122, ThermoFisher) and α-C-kit, 1:250 (AF1356-SP, R&D Systems). For the expression analysis of TAG-NANOS2 and PLZF: α-GFP, 1:500 (GFP-1010, Aves), α-PLZF, 1:100 (sc22839, Santa Cruz Biotechnology). For the expression analysis of TAG-NANOS2 and Dcp1a: α-GFP, 1:500 (GFP-1010, Aves), α-Dcp1a 1:500 (WH0055802M6, Sigma-Aldrich). Secondary antibodies used were all Invitrogen: goat anti-Chicken, Alexa Fluor 488 (A-11039); donkey anti-Rabbit, Alexa Fluor 488 (A-21206); donkey anti-goat, Alexa Fluor 568 (A-11057). Images were acquired by using a Confocal microscope, by taking Z-stacks, and with Z-stepsize set at 0.34 μm. Images were analyzed with Fiji ImageJ. A_s_ and A_pr_ spermatogonia were distinguished by using the 25 μm topographical criteria (Huckins, 1971). If the internuclear distance between two spermatogonia was over 25 μm, cells were assigned to the A_s_ category. On the contrary, spermatogonia whose internuclear distance was smaller than 25 μm, were considered to belong to the same chain.

#### FACS analysis and sorting of SSC lines

For the analysis of GFP expression in SSC lines, MEFs were depleted by a brief sedimentation of MEF clumps and collection of supernatants, which were enriched for SSCs. SSCs were gated from residual MEFs and cell debris by selecting a cell population with low side and forward scatter, by excluding cell doublets, and by selecting only cells negative for the live cell dye DAPI. The analysis was performed with a BD Accuri C6 flow cytometer (BD Biosciences). SSC sorting was performed on FACSAria II Cell Sorter (BD Biosciences) using the same gating strategy detailed above. Data were analyzed using the FlowJo software.

#### CRAC and analysis

The CRAC protocol was performed as described (Granneman et al., 2009; Turowski et al., 2016), with the following modifications. SSCs were grown on feeders in 150 cm^2^ culture dishes for ∼7 days and irradiated with UV-C light (254 nm), with energy set at 0.4 J/cm^2^, using a Stratalinker. TAG-NANOS2 was immunopurified from cell lysate by using an anti-V5 antibody (R960-CUS, Invitrogen) coupled with Dynabeads (11206D, Life technologies). TAG-NANOS2 protein was released from the antibody by proteolytic cleavage, using 40 μg of 3C-GST Protease, at 4°C. Purified libraries were sequenced on an Illumina NextSeq500 instrument, high output 75 bp, single-end run.

Illumina sequencing data were demultiplexed using in-line barcodes and in this form were submitted to GEO. The first quality control step was performed using FastQC software (http://www.bioinformatics.babraham.ac.uk/projects/fastqc/) considering specificity of CRAC data. Raw reads were collapsed to remove PCR duplicates using FASTX-collapser v0.0.14 (http://hannonlab.cshl.edu/fastx_toolkit/) then in-line barcodes were removed using pyBarcodeFilter.py script from pyCRAC package v3.0 (Webb et al., 2014). The 3’ adapters were removed using flexbar v3.4.0 (Dodt et al., 2012) including flexbar quality filter. Pre-processed reads were mapped to the mouse genome (mm10) by using STAR v. 2.5.3a aligner (Dobin et al., 2013) and Salmon v. 0.13.1 quasi-quantification (Patro et al., 2017). Mapped reads were used to determine their distribution among different classes of RNAs using pyReadCounter (pyCRAC package) and HTSeq v.0.11.2 (Anders et al., 2015). BigWig files were generated using bamCoverage v3.1.3 script from deepTools package (Ramírez et al., 2016) and visually inspected. Sam files operations were performed using SAMtools v1.9 (Li et al., 2009).

For the analysis of binding motifs preprocessed *Nanos2*^*TAG/TAG*^ reads were mapped to the mouse transcriptome database generated using Biomart (Smedley et al., 2015). Reads were aligned to the mouse transcriptome using Novoalign v2.07.00 (http://www.novocraft.com) with –r random and saved as sam files. Deletions, reflecting sites of direct RNA-protein contact, were extracted from aligned reads using custom made python script. Further steps were performed for both, full length mapped reads and deletions only. Data were converted to BigWig files using bamCoverage programme. Downstream analysis was performed using python 2.7 Jupiter notebooks, python libraries (pandas v0.19.2, NumPy v1.16.0, scipy v1.2.0) and in-house functions and scripts submitted as an update of gwide toolkit v0.5.27 (https://github.com/tturowski/gwide) (Turowski et al., 2016). Peaks were called for the *Nanos2*^*TAG/TAG*^ profiles (FDR < 0.001) using argrelextrema function from signal processing library scipy.signal (v1.3.0) using order value 20. The total peak score for each gene was calculated by summing the intensities of one or more unique peaks present in each transcript and was expressed as reads per million (rpm) and averaging across two replicate CRAC experiments. These averaged peak scores were then normalized by transcript FPKM (fragments per kilobase million)averaged from two biological replicate mRNA-seq datasets (from *Nanos2*^*TAG/+*^ and *Nanos2*^*TAG/TAG*^ SSC lines). Non-expressed genes, FPKM = 0, were eliminated from the analysis. The resulting ratio values were used to rank the final NANOS2 CRAC list (1428 genes). To define NANOS2 binding site we filtered sequencing reads associated with the top 500 most highly enriched targets, for the ones containing deletions. 3844 reads, corresponding to 498 out of 500 targets, were identified as having deletions. This subset of reads was centered on deletions and only the region spanning 20 or 100 bases around deletions was considered. Motif analysis was performed by using MEME-ChIP (Machanick and Bailey, 2011), and DREME (Bailey, 2011). Centrimo (Bailey and Machanick, 2012) was also used to evaluate the central enrichment of the motifs found. As a background, we used the same sequences shuffled by MEME-ChIP. We looked for motif with E-value < 0.05, width 6-10 bp, with ‘0-1’ or ‘1’ occurrence per sequence, and we scanned only the strand provided.

#### Comparison between CRAC and published RIP datasets

To identify NANOS2 targets from NANOS2 RIP-array experiments in E14.5 male gonads (Saba et al., 2014) and P7 testis (Zhou et al., 2015b), the original array data sets (NCBI GEO accessions GSE37718 and GSE61807) were analyzed. Probe data were background corrected and quantile normalized, followed by fitting of a differential intensity model, using the limma package in R (Ritchie et al., 2015). NANOS2-bound targets were identified as transcripts with a greater than 2-fold excess in signal intensity in the NANOS2 IP versus control IP and by false discovery rate (FDR) of less than 0.05. These target lists were overlapped with our NANOS2 CRAC list. Violin and cumulative distribution plots were generated comparing top-500 NANOS2-bound transcripts (ascertained by normalized CRAC scores) to transcripts not showing NANOS2 binding. Significant differences were detected using Mann-Whitney U tests.

#### RNA isolation and RNA-seq

RNA was isolated from sorted SSCs by using QIAzol Lysis Reagent (79306, Qiagen) and following the manufacturer’s instructions. To generate RNA-seq libraries total RNA was treated with DNAse I, in 10X Buffer (AMPD1, Sigma). RNA was purified using Rneasy MinElute columns (74204, Qiagen) and ribosomal RNA depletion was performed with the RiboCop kit (Lexogen). Ribo-depleted RNA was used to generate libraries with the SENSE Total RNA-Seq Library Prep Kit (Lexogen) following the manufacturer’s instructions. Libraries were sequenced with an Illumina HiSeq2500 on a 50 bp, single-end run.

#### Gene ontology (GO) analysis

The gene ontology (GO) enrichment of the top 500 NANOS2 CRAC targets was determined by using the topGO R package. The enrichment for the biological process ontology was assessed by using Fisher’s exact test (P-value < 0.05).

#### Ingenuity pathways analysis

The pathways enrichment analysis of the top 500 NANOS2 CRAC targets was generated through the use of IPA (QIAGEN Inc., https://www.qiagenbio-informatics.com/products/ingenuity-pathway-analysis) (Krämer et al., 2014). The significance of the enrichment for the signaling pathways was defined by setting -log P-value > 1.3, which corresponds to a 0.05 significance threshold.

#### Immunoprecipitation followed by mass spectrometry (IP-MS)

IP-MS was performed from three replicates of *Nanos2*^*CTL*^ and *Nanos2*^*TAG/TAG*^ SSC lines. TAG-NANOS2 was immunoprecipitated with Anti-GFP antibody (11 814 460 001, Roche) cross-linked to Protein G magnetic beads (88848, Thermo) and processed as described (Much et al., 2016). Peptides were separated on an ultra-high resolution nano-flow liquid chromatography nanoLC Ultimate 3000 unit fitted with an Easyspray (50 cm, 2 μm particles) column coupled to the high resolution/accurate-mass mass-spectrometer Orbitrap Fusion Lumos operated in DDA(data-dependent-acquisition)-mode (Thermo Fisher Scientific). Raw data were processed using MaxQuant version 1.6.1.0. Label-free quantitation (LFQ) was performed using the MaxQuant LFQ algorithm (Cox et al., 2014). Peptides were searched against the mouse UniProt database (date 21.07.2017) with commonly observed contaminants (e.g. trypsin, keratins, etc.) removed during Perseus analysis (Cox et al., 2014; Hubner et al., 2010; Tyanova et al., 2016; The UniProt Consortium, 2017). For visualization, LFQ intensities were imported into Perseus version 1.6.0.2 (Tyanova et al., 2016) and processed as described (Hubner et al., 2010).

#### SLAM-seq

The optimal concentration of 4SU (4-Thiouridine) for SSC lines (100 μM) was determined by using the cell viability titration assay in the Lexogen SLAMseq Explorer Kit. SSCs labeling with 4SU was performed as followed: media with 4SU was supplied twice to SSCs, 24 and 3 hours before time 0 (t_0_). At t_0_, 4SU-media was replaced with 4SU-free-media and SSCs were isolated 0, 30 minutes, 1, 2, 9 and 24 hours later.

1.5 x 10^6^ SSCs were isolated by FACS for each sample and timepoint. SLAM-seq libraries were prepared by using the Lexogen SLAMseq Kinetics Kit – Catabolic Kinetics Module and the Lexogen QuantSeq 3′ mRNA-Seq Library Prep Kit FWD for Illumina, following the manufacturer’s’ instructions. Libraries were sequenced using an Illumina HiSeq2500 platform on a 50 bp, single-end run. Two biological replicates were used to generate libraries with each biological replicate was composed of two technical replicates.

Analysis of SLAM-seq libraries was performed by using the SlamDunk pipeline (Neumann et al., 2019). T to C conversion rates obtained from different time points were normalized to t_0_ for each gene and were used to fit a first-order decay reaction, with the R stats package nls function. The two technical replicates present in each biological replicate were collapsed before calculation of half-life.

### Quantification and statistical analysis

All details of statistical analyses, including replicates, statistical tests and outcomes, are described in the Method details, main results section, and Figure legends.

## Supplemental information

Document S1. Figures S1–S4

Table S1. Results of TAG-NANOS2 CRAC, related to figure 2 The read counts from the TAG-NANOS2 and control CRAC are shown.

Table S2. The top 500 ranked TAG-NANOS2 bound transcripts, related to figures 2 and 3 The top 500 genes as defined by the ratio between the CRAC signal intensity and mRNA abundance.

Table S3. Overlapping genes from figure S3B, related to figure S3 Overlapping genes between the top-500 TAG-NANOS2 CRAC targets from SSC lines and mRNAs identified as NANOS2 targets by RIP from P7 testes (Zhou et al., 2015b).

Table S4. Overlapping genes from figure S3C, related to figure S3 Overlapping genes between the top-500 TAG-NANOS2 CRAC targets from SSC lines and mRNAs identified as NANOS2 targets by RIP from E14.5 male gonads (Saba et al., 2014) are listed.

Table S5. NANOS2-associated proteins in SSC lines, related to figure 4 Proteins found to interact by MS with TAG-NANOS2 in SSC cell lines with a greater than 4 fold enrichment and significance of less than P 0.05 are listed for untreated and RNase-treated IPs.

## Footnotes

Supplemental information can be found online at https://doi.org/10.1016/j.isci.2021.102762.
