# Supplementary material for: NANOS2 is a sequence-specific mRNA-binding protein that promotes transcript degradation in spermatogonial stem cells
Source: iScience. 2021 Jun 24;24(7):102762. doi: 10.1016/j.isci.2021.102762 (PMC8271163; doi:10.1016/j.isci.2021.102762)
Supplement: Document S1. Figures S1–S4 [file mmc1.pdf]

iScience, Volume *24*

Supplemental information

NANOS2 is a sequence-specific mRNA-binding protein that promotes transcript degradation in spermatogonial stem cells

Azzurra Codino, Tomasz Turowski, Louie N. van de Lagemaat, Ivayla Ivanova, Andrea Tavosanis, Christian Much, Tania Auchynnikava, Lina Vasiliauskaitė, Marcos Morgan, Juri Rappsilber, Robin C. Allshire, Kamil R. Kranc, David Tollervey, and Dónal O'Carroll

iScience, Volume --

Supplemental information

NANOS2 is a sequence-specific mRNA-binding protein that promotes transcript degradation in spermatogonial stem cells

Azzurra Codino, Tomasz Turowski, Louie N. van de Lagemaat, Ivayla Ivanova, Andrea Tavosanis, Christian Much, Tania Auchynnikava, Lina Vasiliauskaitė, Marcos Morgan, Juri Rappsilber, Robin C. Allshire, Kamil R. Kranc, David Tollervey, and Dónal O'Carroll

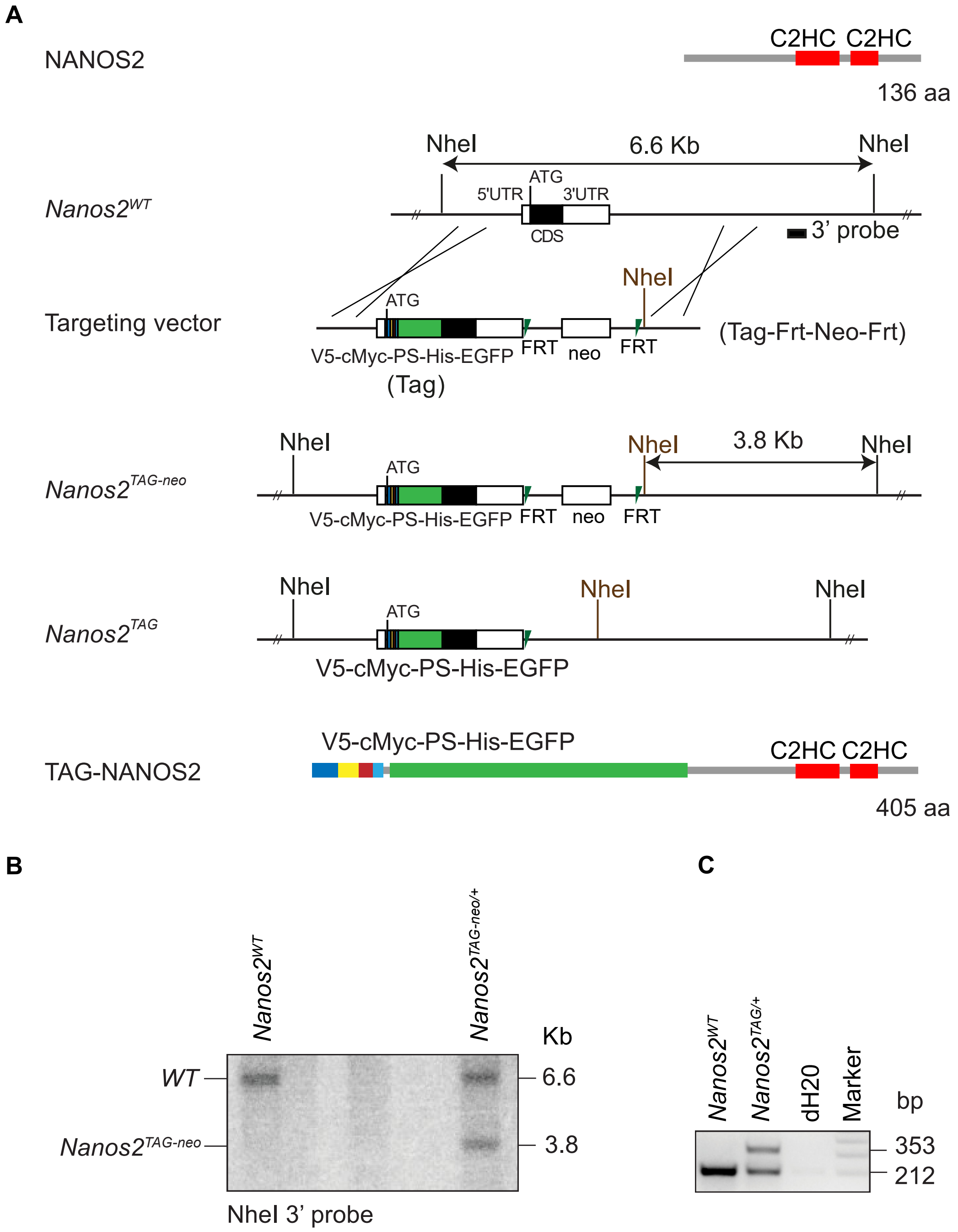

Figure S1. Targeting strategy used for the generation and analysis of the Nanos2^{TAG} mouse allele, Related to Figure 1.

(A) At the top, schematic representation of the Wild-type NANOS2 protein, with the two zinc finger domains (C2HC) in red. Amino acid sequence length (aa) is also shown. Below, the figure shows: the Nanos2^{WT} locus, composed of a protein-coding region (CDS) (black box), 5' and 3' UTR regions (white boxes); the targeting vector, composed of V5-c-Myc-PS-His6X-EGFP (PS is PreScission), for the insertion of the TAG after the first ATG of Nanos2; the targeted Nanos2$^{TAG\text{-}neo}$ locus, before the FLP-mediated deletion of the neomycin (neo) resistance cassette (white box after the 3' UTR), flanked by FRT sites (green triangles). The NheI restriction sites are also shown: in black, NheI sites on the Nanos2^{WT} locus and in brown the ones introduced with the targeting vector. Also, the 3' probe (black box near the Nanos2^{WT} locus) used for Southern blotting and the expected fragments sizes (6.6 Kb, 3.8 Kb) are shown. At the bottom, the structure of the resulting N-terminal TAG-NANOS2 fusion protein is also shown (and represented in scale).

(B) Southern blot performed on NheI-digested genomic DNA from Nanos2^{WT} and Nanos2$^{TAG\text{-}neo/+}$ ESCs clones, hybridized with the 3' probe shown in (A).

(C) Nanos2 PCR-genotyping using tail-biopsies from Nanos2^{WT} and Nanos2$^{TAG/+}$ mice, after inducing FLP-mediated recombination and excision of the FRT-flanked neomycin cassette. The expected size of PCR products is expressed in base pair (bp).

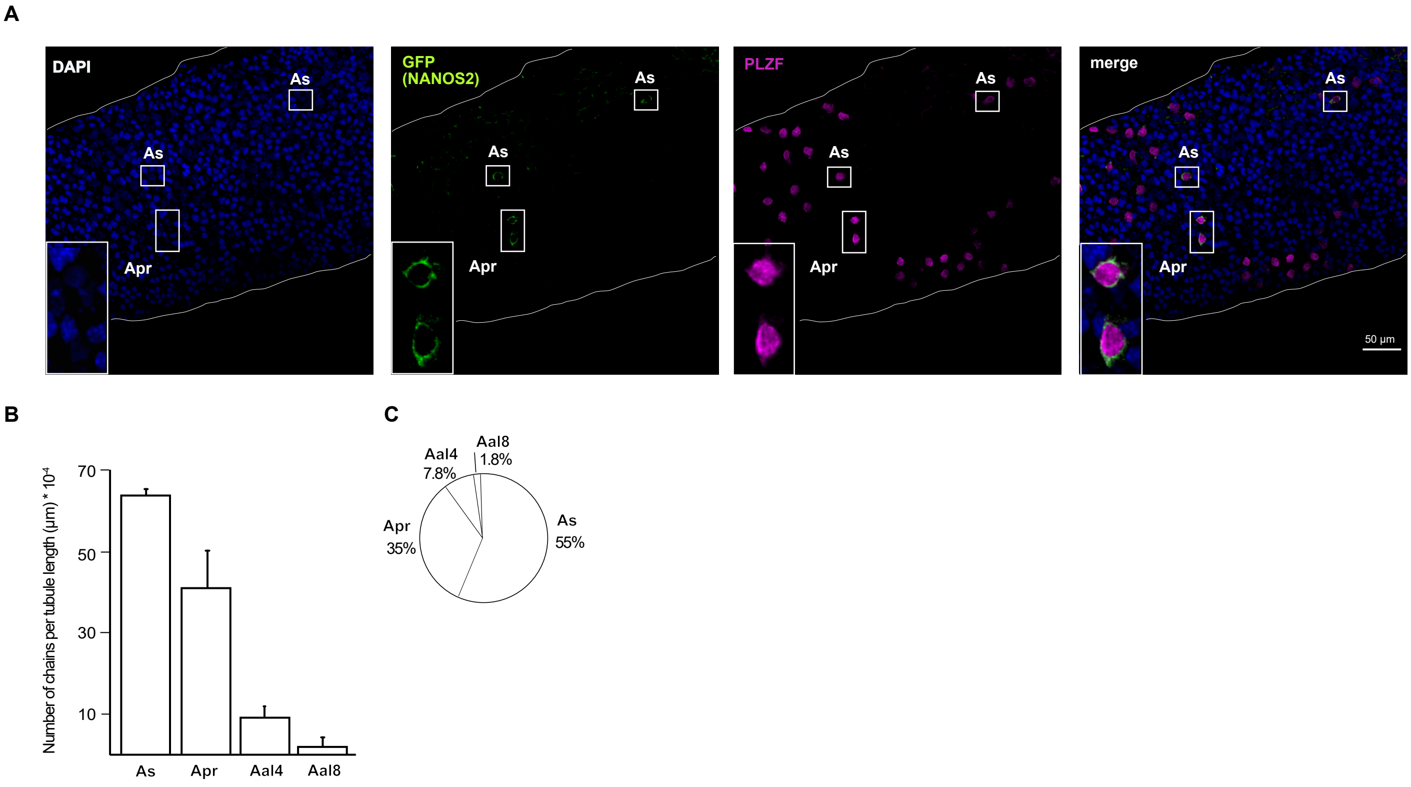

Figure S2. Characterization of TAG-NANOS2 expression in PLZF-expressing spermatogonia, , Related to Figure 1.

(A) Representative immunofluorescent images of *Nanos2*^{TAG} seminiferous tubules stained with DAPI (blue), anti-GFP (green) and anti-PLZF (magenta) antibodies. Representative example of NANOS2^{Pos}, PLZF^{Pos} cell (A_{pr}) is highlighted (white box). Scale bar, 50 µm.

(B) Enumeration of NANOS2^{Pos} cells. On the y-axis, number of chains normalized to tubule length. Error bars represent SEM (standard error of mean).

(C) Pie chart showing the proportion of NANOS2-Positive spermatogonia among different chains: A_s (55%), A_{pr} (35%), A_{al4} (7.8%), A_{al8} (1.8%). All NANOS2^{Pos} cells were found PLZF^{Pos}.

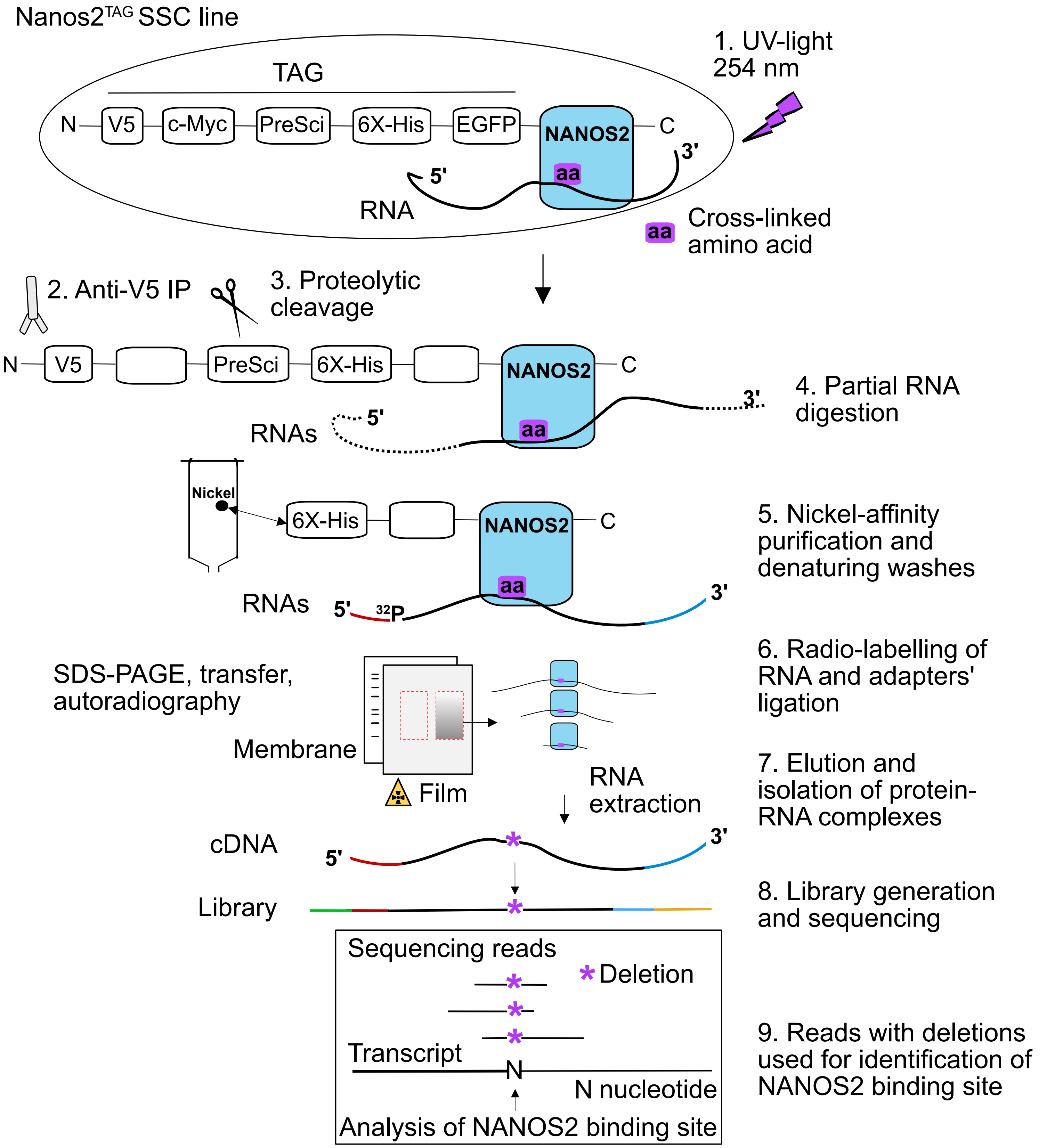

Figure S3. Schematic representation of the experimental procedure used to perform CRAC in *Nanos2*^{CTL} and *Nanos2*^{TAG} SSC lines, Related to Figure 2.
Nanos2^{CTL} and *Nanos2*^{TAG} SSC lines are cross-linked *in vivo* with UV-C light (254 nm), to permanently bind protein-RNA complexes which are at zero-distance (1). Cross-linked amino acid residues (aa) are displayed in purple. After cell lysis, protein-RNA complexes are purified through immunopurification (IP) by using an anti-V5 antibody (2), and by proteolytic cleavage (3). Cleaved protein-RNA complexes are partially digested with RNase (4), and subjected to nickel-affinity purification, which occurs under denaturing conditions (5). Cross-linked RNAs are radiolabelled (^{32}P) and adapters are ligated to the 5' and 3' ends (in red and blue respectively) (6). RNA-protein complexes are eluted from nickel-columns, separated by SDS-PAGE and transferred to a nitrocellulose membrane (7). After visualization of the RNA-protein complexes by autoradiography, these are excised from the membrane. Proteins are digested and RNA is extracted (7). RNA is reverse transcribed, and the resulting cDNA is amplified by PCR to generate libraries for sequencing (8). NANOS2 targets are identified through bioinformatic analysis of sequencing reads (9). Reads with deletions (purple asterisk) are used to identify NANOS2 binding site (N, nucleotide) on the corresponding transcript (9).

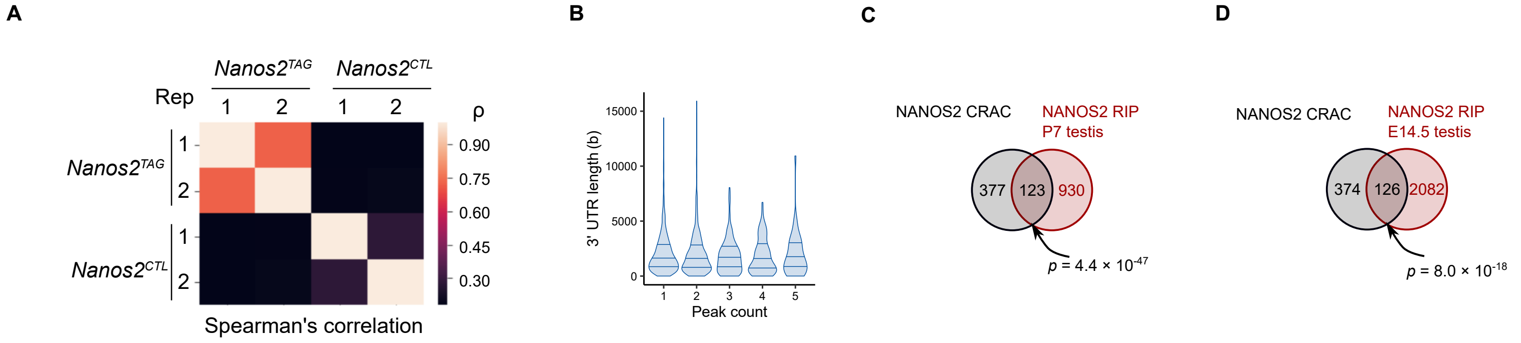

E

Input sequence (size)	Logo	Positives	Negatives	P-value	E-value	Centrimo
Deletions (100 b)	MEME-ChIP DREME 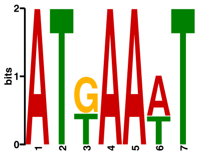	1212/3844	617/3844	4.4e-058	1.8e-053	8.9e-057 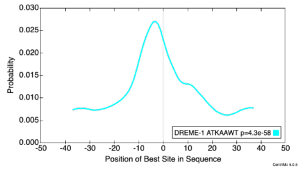

Figure S4. Spearman's correlation for CRAC samples and de novo motif analysis, Related to Figure 2.

(A) Heatmap showing Spearmann's correlation coefficient (ρ) among replicate *Nanos2*^{CTL} and *Nanos2*^{TAG} samples. ρ=1 means perfect positive correlation (faint pink), ρ=0 means no correlation (black).

(B) Violin plots of 3' UTR length for transcripts harbouring indicated numbers (1-5) of NANOS2 CRAC peaks. 2484 NANOS2 CRAC peaks in 1813 unique transcripts (corresponding to 1594 protein-coding genes) showed almost no correlation with transcript length (Pearson R = -0.052, p = 0.026).

(C) Venn diagram showing the overlap between the top-500 TAG-NANOS2 CRAC targets from SSC lines and mRNAs identified as NANOS2 targets by RIP from E14.5 male gonads (Saba et al., 2014). *P*-value of overlap significance was calculated by Fisher's exact test.

(D) Venn diagram showing the overlap between the top-500 TAG-NANOS2 CRAC targets from SSC lines and mRNAs identified as NANOS2 targets by RIP from P7 testes (Zhou et al., 2015b). *P*-value of overlap significance was calculated by Fisher's exact test.

(E) Details of the DREME-MEME *de novo* motif analysis performed on *Nanos2*^{TAG} CRAC sequencing reads containing deletions, associated with the top 500 targets.
